# Doubting doubly labeled water

**DOI:** 10.1073/pnas.2520143122

**Published:** 2025-11-03

**Authors:** Chika Edward Uzoigwe

**Affiliations:** ^a^Department of Surgery, Medicine and Science, Harcourt House, Sheffield S10 1DG, United Kingdom

McGrosky et al. make extensive use of Doubly labeled water (DLW) (1), regarded as a Gold Standard for elucidating energy expenditure and body composition (2). It relies upon the differential clearance of deuterium (^2^H) and oxygen (^18^O) isotopes in labeled water, via fluids and expiration (3). It was conceived in the 1950s and presumed safe as the isotopes are not radioactive (4). At that time, however, there was a gross underestimation of the sophistication of natural selection/biological systems, which can in actuality distinguish between isotopes, preferentially selecting lighter isotopes, which undergo reactions more rapidly. This is isotope fractionation and accounts, for example, for the depletion of ^13^C in plants by up to 20% compared to the atmosphere (5). The greater the difference in isotopic mass, the more profound is this effect. There is 100% difference in mass between hydrogen (^1^H) and deuterium (^2^H), creating a huge biobias toward the former over the latter. By contrast, metabolism, while still distinguishing between ^18^O and ^16^O, is much more permissive of ^18^O compared to deuterium, due to the comparatively modest 12.5% mass difference between the two oxygen isotopes. This fundamentally undermines the very premise by which DLW operates; namely, that the bio-posture toward deuterium is intrinsically the same as that to ^18^O. Simply put biophysically the difference between ^18^O and ^16^O is not the same as that between ^1^H and ^2^H.

Extreme isotope fractionation has been shown in humans following the administration of DLW. Wong et al. reported plasma, urine, saliva, vapor heavy isotope depletion of 14%, 16%, 9%, and 62% for deuterium but 1%, 2%, 1%, and 12.5%, respectively, for ^18^O compared to the composition of DLW consumed (6). Aquaporins prefer unlabeled water, then ^1^H^2^HO and finally ^2^H_2_O. The permeability of deuterated water (^2^H_2_^16^O) through aquaporin is 15 to 25% that of water (7). In addition, smaller/lighter water isoforms will diffuse more rapidly and be absorbed. DLW-based techniques on estimating body fat may thus underestimate the true value. Wang showed carbon dioxide to be enriched with ^18^O at +38%. Thus, the letter is preferentially excreted than incorporated into biomolecules (6).

Host dedication to the elimination of deuterium is founded on the primacy of the proton (^1^H^+^) in the most important and fundamental processes, such as chemiosmosis. These bioprocesses rely on the diminutive size of the proton to exploit rapid classical chemo-kinetics and Nuclear Quantum Effects (8). These essential pathways have been shown to be effectively arrested by substitution with the ponderous, unwieldy, cumbersome heavy-weight deuterium ion. Hence, biosystems deplete the body of deuterium much more aggressively than they do of ^18^O. Some mammals metabolically exclude deuterium by enriching proline with this isotope and then sequestering it within inert integuments such as collagen in fur, nails, teeth, and bone which are ultimately shed (9).

Finally isotopes do not merely cause harm by radioactivity. Studies consistently and reproducibly show that deuterium-depleted water is salubrious, inter alia inhibiting tumor growth (10). It cannot be assumed DLW, deuterium-enriched water is wholly safe or reliable.
